# Smartphone-Driven Low-Power Light-Emitting Device

**DOI:** 10.1155/2017/5076965

**Published:** 2017-04-13

**Authors:** Hea-Ja An, Kyung-Won Kim, Mun-Ho Ryu, Han-Yeong Oh, Nam-Gyun Kim, Kyoung-Jun Park

**Affiliations:** ^1^Department of Healthcare Engineering, Chonbuk National University, 567 Baekje-daero, Deokjin-gu, Jeonju-si 54896, Jeollabuk-do, Republic of Korea; ^2^Division of Biomedical Engineering, Chonbuk National University, 567 Baekje-daero, Deokjin-gu, Jeonju-si 54896, Jeollabuk-do, Republic of Korea; ^3^Research Center of Healthcare & Welfare Instrument for the Aged, Chonbuk National University, 567 Baekje-daero, Deokjin-gu, Jeonju-si 54896, Jeollabuk-do, Republic of Korea; ^4^Medical Research Center, Color Seven Co., Ltd., Seoul, Republic of Korea

## Abstract

Low-level light (laser) therapy (LLLT) has been widely researched in the recent past. Existing LLLT studies were performed based on laser. Recently, studies using LED have increased. This study presents a smartphone-driven low-power light-emitting device for use in colour therapy as an alternative medicine. The device consists of a control unit and a colour probe. The device is powered by and communicates with a smartphone using USB On-The-Go (OTG) technology. The control unit controls emitting time and intensity of illumination with the configuration value of a smartphone application. Intensity is controlled by pulse width modulation (PWM) without feedback. A calibration is performed to resolve a drawback of no feedback. To calibrate, intensity is measured in every 10 percent PWM output. PWM value is linearly calibrated to obtain accurate intensity. The device can control the intensity of illumination, and so, it can find application in varied scenarios.

## 1. Introduction

Recently, low-level light (laser) therapy (LLLT), a kind of colour therapy, has been widely studied. LLLT has clinical applications with low-emitting laser or light-emitting diode (LED). Effects of LLLT are shown in molecular, cellular, and tissue levels. It has positive effects to promote healing and to relieve some pain or inflammation. The biological basis mechanism of LLLT is not clear. It is thought that red or near infrared (NIR) light is absorbed by mitochondrial chromophores and also by photoreceptors in the plasma membrane of the cells. Many studies have assumed that some biological action is caused by subsequent reactions after that [1].

Existing LLLT studies have been performed based on laser. Studies with laser LLLT have researched the effects of laser in cell growth causing recovery from wound [2]. Rizzi et al. have studied the effects of LLLT on traumatized muscle repair process. They have tracked the nuclear factor NF-*κ*B and measured its activity. Ga-As laser (904 nm, 45 mW, and 5 J/cm^2^) is used, and the results have shown a decrease in inflammation on traumatized muscle [3]. Landucci et al. have researched LLLT effects on randomly selected patients having left or right third molar extraction operation. Ease from pain and swelling is estimated by a score of visual analogue scale after the operation. The light-emitting 10 mW is used and 780 nm wavelength is selected, as infrared LLLT effectively penetrates the deeper tissue. Furthermore, not only intraoral applications but also extraoral applications of LLLT appear to reduce pain and swelling after surgery [4].

Recently, studies using LED have increased instead of researches focusing on laser therapy. The effect of LLLT on cellular function has been demonstrated. Nishioka et al. have researched using LED LLLT on a skin flap commonly used in plastic surgery. During the recovery period of the skin flaps, necrosis of the flap usually occurs. This LED LLLT is used to control necrosis and promote growth of mast cells and angiogenesis. Four groups were tested, and 660 nm laser and 630 nm LED were used for the experiment. The result of the comparison between the laser and LED has shown that the LED therapy is more effective [5]. Using LED LLLT is effective in releasing muscle fatigue after exercise. LED LLLT (830 nm, 200 mW, and 30 J) with a multidiode cluster was performed 5 minutes before running, and it was found that oxidative stress was reduced. This means that muscle fatigue and damage were decreased [6].

Lately, device development based on smartphones has been actively studied. For example, a sensor with an internal battery is implemented to communicate with the smartphone using Bluetooth or Universal Serial Bus (USB) [7]. Huang et al. have developed a mobile device to detect blood flow using an ultrasonic sensor. The mobile device detects the sound of blood flow using the Doppler effect. The smartphone can get the signal through its audio line, and the ultrasonic signal is processed using a smartphone application [8]. Muramatsu and Sasaki have studied lighting colour control system using a smartphone. The electrode is connected using the USB. After the colour is picked on the smartphone application, a user touches the other side of the electrode and the lighting colour is changed [9].

This study presents a mobile LLLT device controlled by a smartphone using USB On-The-Go (OTG). The LLLT effect is acquired by LED lighting, and the device is controlled by a smartphone application. The USB provides a communication between the device and the smartphone. Especially, the smartphone also provides power to the device via the USB OTG, which is a unique idea of the proposed system. The device has 610 nm wavelength to perform colour therapy [10]. The device acts as a USB peripheral. To control LED, field-effect transistor (FET) open-loop circuit is selected for simplicity and ease of development. Since the FET open loop has no feedback circuit, there is inaccuracy in LED lighting according to the variations in the LED and circuit components [11]. To compensate for this inaccuracy, LED brightness is calibrated on every probe. Effectiveness of calibration is tested by using Z-statistic.

## 2. Materials and Methods

**Figure 1:**
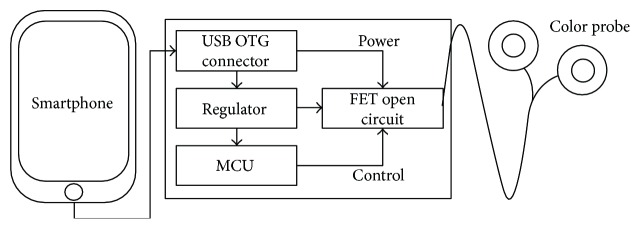
Device elements.

**Figure 2:**
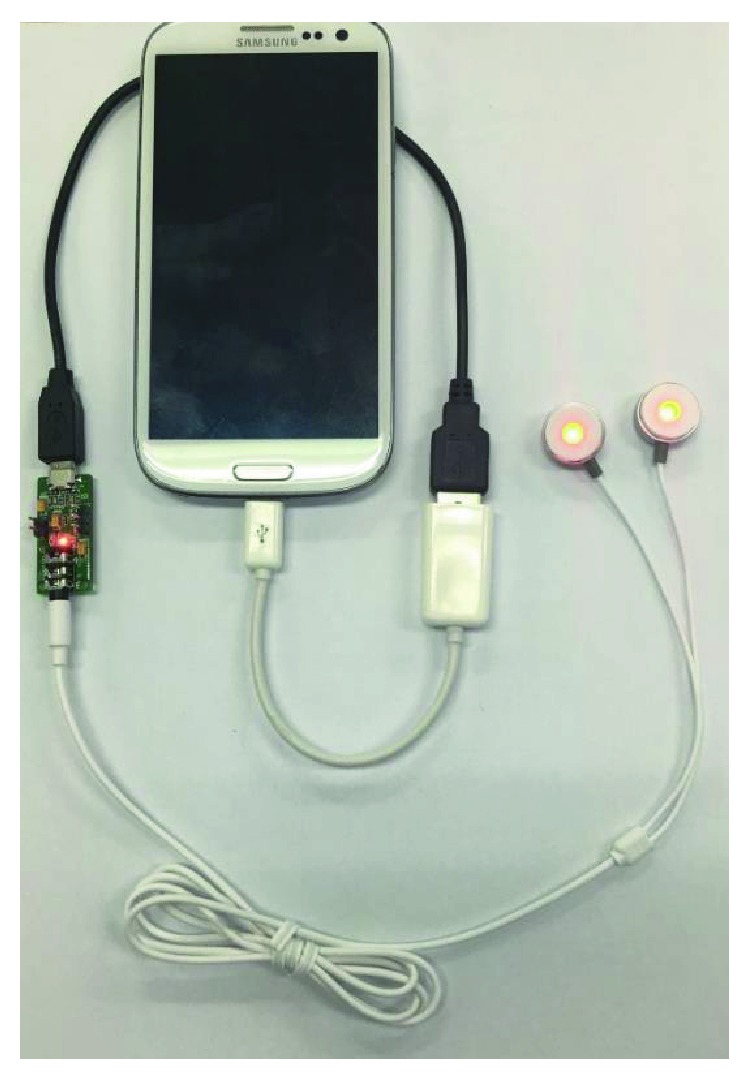
Device setup.

The device consists of a control unit and a colour probe. A smartphone application controls the LED brightness and operating time. The USB OTG is used not only for providing a communication line but also for providing power supply to the device. The smartphone application sends the intended brightness of LED and operating time through the USB, and the data are processed by a microcontroller unit (MCU) in the control unit. The smartphone provides 5 V, of which 3.3 V is needed for use in MCU. So, a regulator circuit is implemented and adequate voltage is supplied to the MCU. A power switch with FET in open-loop form is implemented for ease and simplicity. Probe unit is made of LED-lighting module for ease of use (Figures 1 and 2).

This study uses USB OTG to connect the smartphone and the LLLT device. The used MCU also provides API, and communication can easily be implemented. The USB OTG was first introduced in 2001, and it helps connect devices, for example, smartphone to the peripheral device, like a mouse or keyboard. When the two devices are connected, one part of the controlling communication line is called master and the other, slave. In this system, the smartphone is used as master and the LLLT device is used as a slave.

**Figure 3:**
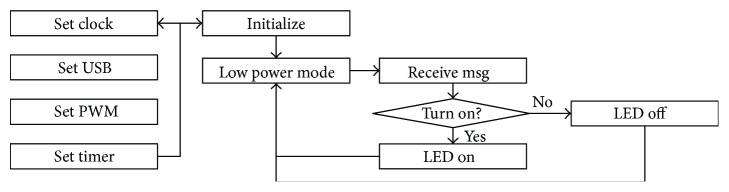
Firmware flow chart.

The MCU used in this study is MSP430F5502 (Texas Instruments, TX). This MCU has a characteristic of low-power consumption, and it generally uses 195 uA/MHz in active state. The LLLT device performs the functions using the smartphone battery, and so, the current consumption would be adequate. The clock of the MCU is configured for 4 MHz. USB communication and PWM are configured, respectively. After initialization, the MCU enters low-power mode and waits for messages from the smartphone application. The MCU receives and decodes messages and gets proper actions (Figure 3).

There are several methods to control LED brightness, such as PWM dimming, frequency modulation dimming, and analogue dimming [11]. The PWM dimming has the strength to stabilize the wavelength if duty cycle is changed. However, the brightness cannot be linearly controlled within 10%. The frequency modulation dimming has multiple frequency spectra and is used to decrease electromagnetic interference noise. In case of lengthening low-level duty cycle, even the blink of our eyes can be detected. The analogue dimming method controls the current value that flows to the LED using a feedback circuit, so the brightness is controlled. However, the wavelength is unstable. The PWM dimming has a drawback that it is unstable at low levels. However, the device has no need to be controlled under 10%, and so, the PWM dimming is the preferred method for the study considering the ease and simplicity of implementation.

The MCU sets up the brightness value of colour probes through PWM signal. PWM is a method to convert a digital signal to a pseudoanalogue one. In an output time of a signal's high state, that is, duty cycle is adjusted to control output voltage signal [11]. On the other hand, output current from MCU is up to 8 mA which is not enough to light the LEDs. So, a FET open circuit is built to supply the necessary current. The power source of the FET open circuit is the smartphone battery, so enough current can be provided to the LEDs.

**Figure 4:**
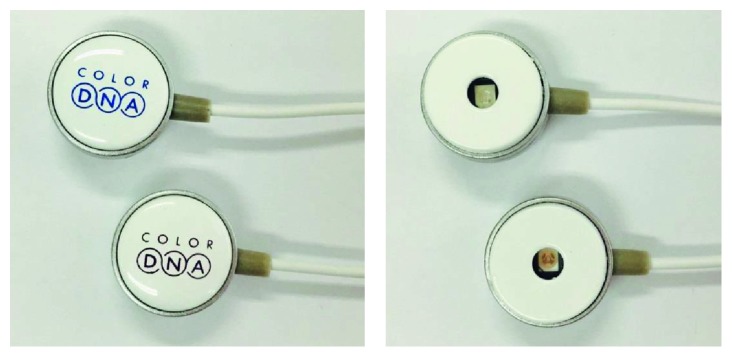
Probe top side (left) and probe bottom side (right).

The probe unit includes the LEDs (Ciel Light, Korea) with 610 nm wavelength. Its size is 16 mm × 8 mm and rated current is 20 mA. The LED module has the form of earphones (Figure 4). They are attached to the skin with medical-grade double-sided tape for biocompatibility. The 610 nm wavelength has been adopted as the LLLT in this study [10]. Light energy for lighting skin is about 1 J/cm^2^. At maximum brightness, the rated current of the colour probes is 20 mA; a treatment cycle is set to twenty minutes. Therefore, they consume 14 mAh in a cycle. With a smartphone battery of 2000 mAh capacity, the LLLT device can be used about 140 times.

## 3. Experiment

**Figure 5:**
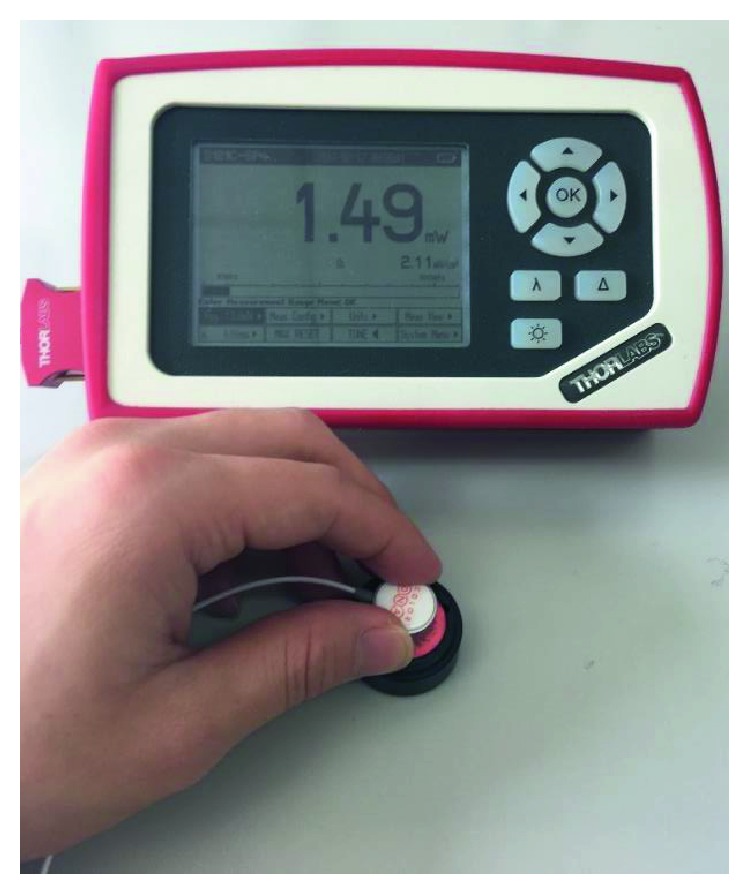
Brightness measurement equipment.

To measure the LED brightness, a compact power and energy meter console (PM100D, Thorlabs Inc., NJ) was used as a test equipment. The equipment can capture wavelength between 400 nm and 1100 nm. Its measurable range of power is between 500 nW and 500 mW with 3% measurement uncertainty. It has a 10 nW resolution (Figure 5).

**Figure 6:**
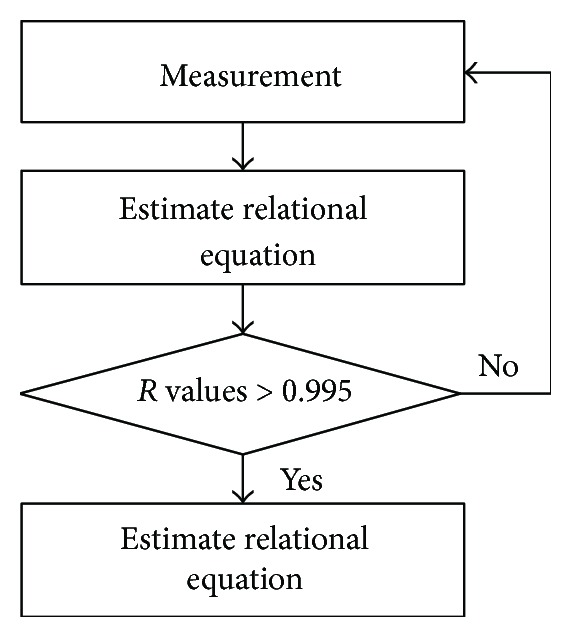
Calibration algorithm.

The LED brightness is checked to estimate brightness-PWM relational expression. The test is performed 5 times on each probe. Totally, there are 1000 times tests; 20 probes (10 probe pairs), 5 times repetition, and 10 steps on every 10%. The expression is estimated with the collected data. The expression is calculated for linear functions with method of linear squares; the correlation coefficients of each linear function are also calculated. If the calculated coefficients are lower than 0.995, the result function is discarded because the function is not judged to have a similarity with the test results. Then, the reverse function is calculated (Figure 6). The calibrated PWM values for lighting are taken from the reverse function.

For analysing calibration results statistically, the Z-statistic is chosen. The Z-statistic is a statistical method to analyse that a population and a sample have a significant difference. Using the Z-statistic, the confidence interval is calculated. The interval is from (0.15 − (1.96 × SEM)) to (0.15 + (1.96 × SEM)), where SEM is the standard error of the mean. The SEM value is the standard deviation divided by root square of the sample.

## 4. Results

**Figure 7:**
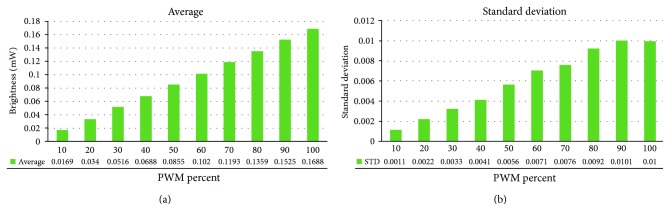
(a) Brightness averages and (b) standard deviations.

10 LED probe pairs have been tested, and it was found that each has a different average brightness between 0.153 mW and 0.180 mW (Table 1). The standard deviations of brightness tend to increase, and the biggest deviation, 0.0094 mW, is shown at 100%. Brightness and the standard deviations increased almost linearly (Figure 7). Therefore, the relational expression between brightness and PWM is calculated by a linear square method. Then, correlation coefficients can be calculated, and all probes are found to have a score more than 0.995.

**Figure 8:**
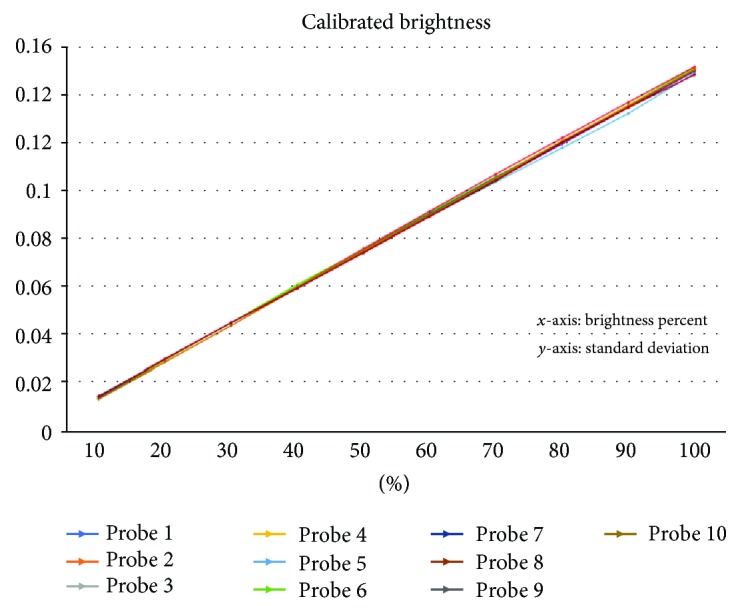
Calibrated brightness standard deviations.

The reverse expression has been calculated to get the calibrated PWM input values using the calibration algorithm explained just before. The calibrated PWM values are accumulated on a table, and the brightness is tested again in the same way as before. It is measured 0.15 mW based on 100% PWM. After calibration, standard deviations are collected between 0.0004 mW and 0.0012 mW (Figure 8). The standard deviation on 100% PWM is decreased from 0.01 mW to 0.0012 mW.

**Figure 9:**
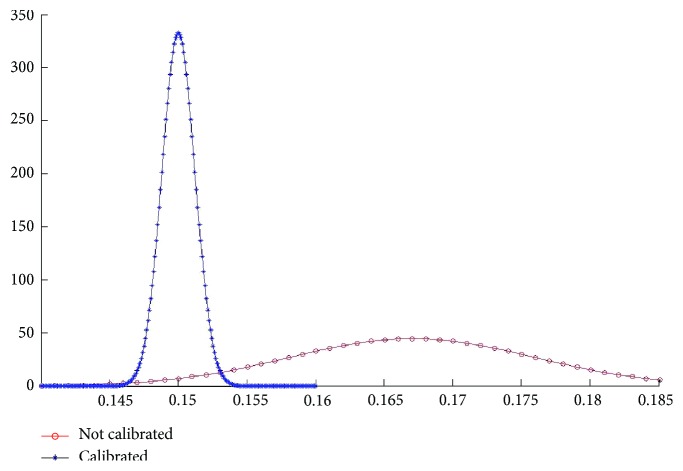
Normal distribution of measurement.

Before calibration, the average of brightness is found to be 0.166 mW and the standard deviation is 0.0094 mW. And after calibration, the average is 0.150 mW and the standard deviation is 0.0012 mW (Figure 9).

## 5. Discussion

This study has adopted FET open circuit for simplifying the development of the LLLT device. The absence of feedback loop means the FET open circuit cannot provide stability and accuracy of the device. Each probe has different values of brightness. However, they have a tendency to linearly change. So, the linear calibration algorithm can be implemented and properly tested. After the device was configured, emitting light is estimated by using the measuring instrument. The test was performed for 10 probes and 5 times on each of them. After the device has been calibrated, stability in brightness can be achieved by decreasing standard deviations.

In the study, the brightness objective is 0.15 mW and the standard deviation is 0.006. The result is verified by using Z-statistic when the population mean and standard deviation are known. To analyse calibration statistically, the confidence interval to brightness is calculated from 0.144 mW to 0.155 mW with the Z-statistic. After calibration, every result on 100% is within this targeted interval. Standard deviation is seen to decrease from 0.01 mW to 0.0012 mW. The control of brightness on FET open circuit becomes stable with use of calibration algorithm.

## 6. Conclusion

This study presents that an LED LLLT device is developed using a widely available smartphone. The main idea and originality of this study is to propose a smartphone-driven LED LLLT device that is controlled by and powered by a smartphone. Generally, LLLT device is composed of a power source, a controller, and emitting modules usually, so it is not easy to carry. The device resolves this problem because it is developed compactly and friendly. The device has no need for any other battery or a controller because the smartphone performs as a power source and a controller. USB OTG was used to provide power supply and communication with a smartphone application. Simultaneously, the FET open circuit was implemented for ease of development and small size. A calibration algorithm was implemented to solve stability and accuracy issues, which are drawbacks of the FET open circuit. The algorithm was verified through tests. The measurement results were within the targeted acceptable range, 0.144 mW to 0.155 mW. The interval is calculated and confirmed with the Z-statistic. No significant difference from expectation was observed. This means that the algorithm has performed as expected and the developed device is controlled accurately.

## Conflicts of Interest

The authors declare that they have no conflicts of interest.

## Figures and Tables

**Table 1:** Brightness average of each probe (in mW).

LED probes	Brightness percent									
	10%	20%	30%	40%	50%	60%	70%	80%	90%	100%
Probe 1	0.015	0.031	0.046	0.063	0.076	0.089	0.107	0.122	0.134	0.153
Probe 2	0.017	0.033	0.050	0.066	0.081	0.098	0.114	0.130	0.144	0.165
Probe 3	0.016	0.032	0.048	0.064	0.081	0.096	0.112	0.127	0.144	0.156
Probe 4	0.017	0.035	0.051	0.068	0.084	0.101	0.117	0.134	0.152	0.167
Probe 5	0.018	0.036	0.053	0.071	0.089	0.107	0.125	0.143	0.158	0.172
Probe 6	0.018	0.036	0.055	0.072	0.089	0.107	0.123	0.142	0.162	0.179
Probe 7	0.018	0.036	0.054	0.071	0.090	0.106	0.126	0.141	0.159	0.175
Probe 8	0.018	0.037	0.055	0.074	0.092	0.110	0.128	0.147	0.163	0.180
Probe 9	0.016	0.032	0.055	0.074	0.092	0.110	0.128	0.147	0.163	0.180
Probe 10	0.016	0.032	0.049	0.065	0.081	0.096	0.113	0.126	0.146	0.161
